# Single-cell RNA sequencing uncovers heterogenous transcriptional signatures in macrophages during efferocytosis

**DOI:** 10.1038/s41598-020-70353-y

**Published:** 2020-08-31

**Authors:** Connor Lantz, Behram Radmanesh, Esther Liu, Edward B. Thorp, Jennie Lin

**Affiliations:** 1 Department of Pathology, Pediatrics, Medicine and the Feinberg Cardiovascular and Renal Research Institute, Feinberg School of Medicine, Northwestern University, Evanston, USA; 2 Jesse Brown Veterans Affairs Medical Center, Chicago, IL 60611 USA

**Keywords:** Cell death and immune response, Innate immunity

## Abstract

Efferocytosis triggers cellular reprogramming, including the induction of mRNA transcripts which encode anti-inflammatory cytokines that promote inflammation resolution. Our current understanding of this transcriptional response is largely informed from analysis of bulk phagocyte populations; however, this precludes the resolution of heterogeneity between individual macrophages and macrophage subsets. Moreover, phagocytes may contain so called “passenger” transcripts that originate from engulfed apoptotic bodies, thus obscuring the true transcriptional reprogramming of the phagocyte. To define the transcriptional diversity during efferocytosis, we utilized single-cell mRNA sequencing after co-cultivating macrophages with apoptotic cells. Importantly, transcriptomic analyses were performed after validating the disappearance of apoptotic cell-derived RNA sequences. Our findings reveal new heterogeneity of the efferocytic response at a single-cell resolution, particularly evident between F4/80^+^ MHCII^LO^ and F4/80^−^ MHCII^HI^ macrophage sub-populations. After exposure to apoptotic cells, the F4/80^+^ MHCII^LO^ subset significantly induced pathways associated with tissue and cellular homeostasis, while the F4/80^−^ MHCII^HI^ subset downregulated these putative signaling axes. Ablation of a canonical efferocytosis receptor, MerTK, blunted efferocytic signatures and led to the escalation of cell death-associated transcriptional signatures in F4/80^+^ MHCII^LO^ macrophages. Taken together, our results newly elucidate the heterogenous transcriptional response of single-cell peritoneal macrophages after exposure to apoptotic cells.

## Introduction

The clearance of apoptotic cells, termed efferocytosis^1^, is executed billions of times each day by a diverse spectrum of macrophage (MΦ) subsets in humans^2^. This process induces an active mRNA transcriptional response within the phagocyte that is necessary to maintain tissue homeostasis and a nonphlogistic milieu^3,4^. Additionally, efferocytosis within an injured tissue promotes the secretion of anti-inflammatory cytokines^5^ and pro-resolving mediators^6^ contributing to protective responses during tissue repair^7^. However, impaired efferocytosis contributes to failed inflammation resolution and therefore is a significant feature of chronic inflammation diseases^8^*.* For instance, in advanced atherosclerosis defective efferocytosis induces plaque necrosis and inflammation leading to subsequent plaque disruption and thrombosis^9,10^.

Genetic fate-mapping combined with parabiosis and adoptive transfer approaches have revealed that macrophage diversity may originate from two broad ontogenetic categories^11,12^. This includes (1) embryonic precursors that differentiate into self-renewing tissue-resident macrophages and (2) bone marrow-derived haematopoietic stem cells (HSCs) that differentiate into blood monocytes^11^. Specifically within the peritoneum, Cd11b^+^ F4/80^+^ large peritoneal macrophages (LPMs) appear to be embryonically-derived, while Cd11c^+^ MHCII^+^ small peritoneal macrophages (SPMs) are sourced from adult blood monocytes^13,14^. Cd11b^+^ F4/80^+^ LPMs are dependent on the transcription factor GATA-binding factor 6 (GATA6) for homeostatic cellular maintenance^15^, while Cd11c^+^ MHCII^+^ SPMs require IRF4 for differentiation from monocytic precursors highlighting disparities among peritoneal macrophage subsets^13^. Moreover, these distinct populations rely on disparate molecular mediators to conduct and respond to efferocytosis^16^. Consequently, these MΦ populations would display heterogeneous transcriptional signatures that could not be assigned to phagocyte subpopulations after quantitative PCR or bulk sequencing of mixed macrophage cultures. Indeed the transcriptomics of bulk macrophages during efferocytosis has been characterized in detail^4,17^. Such analyses have defined conserved activation profiles including mobilization of the cytoskeletal network and transcriptional induction of target mRNAs by nuclear receptors, such as Liver X Receptor (LXR)^18^. Yet, our appreciation of how distinct phagocyte subpopulations may uniquely reprogram in response to apoptotic cells has not yet been resolved at the single cell level.

Herein, we describe the transcriptomic signature of primary single cells during efferocytosis and after exposure to apoptotic cells. To control for apoptotic cell-derived transcripts^19^, primary macrophages were co-cultivated with apoptotic cells of a separate species. With this experimental design, we are able to discriminate mRNAs derived from the phagocyte versus mRNAs derived from apoptotic cells. In addition, apoptotic cells that expressed a transgenic reporter gene were utilized as a supplemental reporter to track apoptotic cell catabolism. Thus, our results newly elucidate the heterogenous transcriptional response of single-cell peritoneal macrophages, after exposure to apoptotic cells. These data and analyses will be useful for future investigations that seek to determine the unique functional roles of phagocyte subsets during efferocytosis and after exposure to apoptotic cells.

## Results

### Single-cell mRNA sequencing reveals distinct resident peritoneal macrophages at steady state

**Figure 1:**
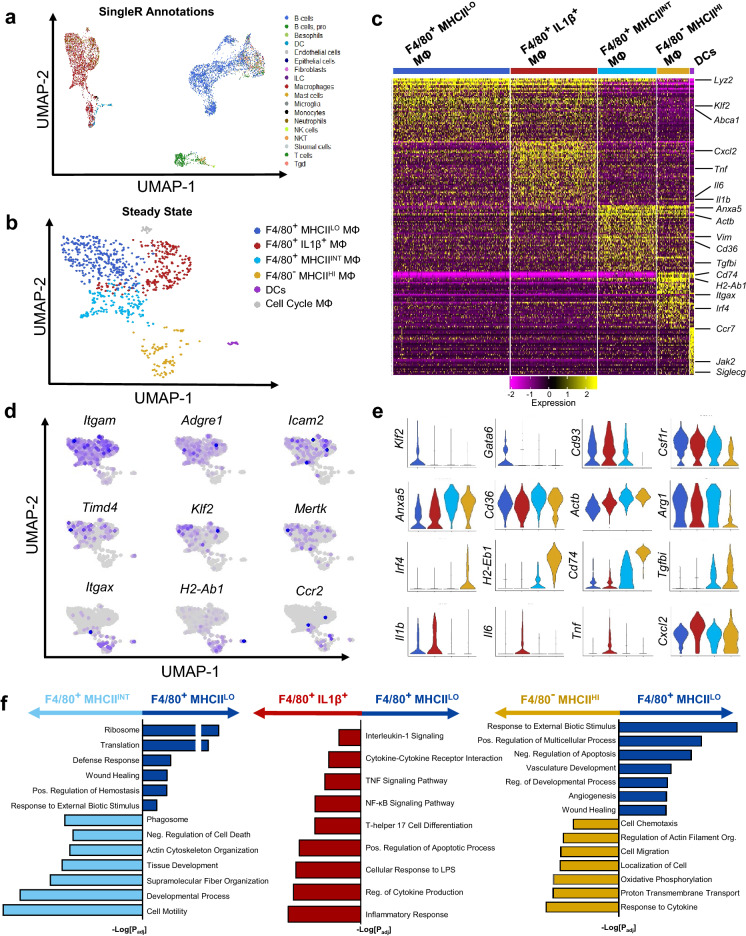
Single-Cell RNA Sequencing reveals distinct resident peritoneal macrophages at steady state. Resident peritoneal macrophages were isolated from C57BL/6 J mice (n = 3) after lavage. Transcriptomic analysis was performed on 1,430 individual cells using the 10 × Genomics platform. (**a**) Unbiased assignment of peritoneal immune cell identity using SingleR and Immgen’s reference database. (**b**) High-Resolution UMAP dimensional reduction of macrophage and dendritic cell (DC) partitioned into 5 distinct clusters. (**c**) Heatmap displaying the top 50 most upregulated gene in each cluster defined in (b). (**d**) Gene expression patterns representing single-cell gene expression of canonical peritoneal macrophage markers. (**e**) Violin plots of cluster-defining genes revealing distinct expression of macrophage differentiation mediators and inflammatory genes. (**f**) Differentially expressed genes in the F4/80^+^ MHCII^LO^ cluster were compared to the F4/80^+^ MHCII^INT^ cluster, the F4/80^−^ IL1β^+^ cluster and the F4/80^−^ MHCII^HI^ cluster using gProfiler. Pathway enrichment is expressed as the –log[p.value] adjusted for multiple comparisons.

To first define resident peritoneal immune cell heterogeneity, we isolated cells from adult C57BL/6J mice after peritoneal lavage and performed single-cell mRNA sequencing (scRNA-seq) on 1,430 individual cells using the 10 × Genomics platform. We employed uniform manifold approximation and projection (UMAP) dimensionality reduction analysis combined with unbiased cell type recognition using the Immgen^20^ open-source reference database to identify populations consisting of murine MΦs, B and T lymphocytes, and dendritic cells (DCs) (Fig. 1A, Supplemental Fig. S1). Higher resolution clustering using UMAP dimensionality reduction identified 4 discrete MΦ subsets and 1 DC population (Fig. 1B,C). As introduced earlier, both traditional LPMs (Cd11b^+^F4/80^+^) and SPMs (Cd11c^+^ MHCII^+^) are represented in the dataset. Specifically, cluster 1, 2, and 3 are enriched for *Itgam* (Cd11b) and *Adgre1* (F4/80) expression, while cluster 4 is enriched for *Itgax* (Cd11c), *Ccr2* (CCR2)*,* and *H2-Ab1* (MHCII) expression (Fig. 1D). Additionally, efferocytic receptors *Mertk* and *Timd4* along with *Klf2*, a transcription factor that controls the expression of many components of the AC clearance program^21^, are enriched in cluster 1, 2, and 3 (Fig. 1D). Further characterization of each cluster revealed distinct expression of macrophage differentiation mediators. Cluster 1 (termed F4/80^+^ MHCII^LO^ MΦs) expressed genes such as *Gata6* and *Klf2* (Fig. 1E). Cluster 2 (termed F4/80^+^ MHCII^INT^ MΦs) had higher *Anxa5, Cd36,* and *Actb* expression. Cluster 3 (termed F4/80^+^ IL1β^+^ MΦs) was enriched in many inflammation-stimulated genes such as *Il1b*, *Il6,* and *Tnf* (Fig. 1E). Lastly, cluster 4 (termed F4/80^−^MHCII^HI^ MΦs) expressed components of the MHCII complex such as *Cd74* and *H2-Ab1* while also expressing *Irf4*, a critical transcription factor for monocyte-derived peritoneal MΦ development^13^ (Fig. 1D,E).

We next performed pathway analysis by directly comparing differentially expressed genes between the F4/80^+^ MHCII^LO^ MΦs to other clusters individually. Compared to F4/80^+^ MHCII^LO^ MΦs, F4/80^+^ MHCII^INT^ MΦs were enriched for pathways associated with efferocytosis such as phagosome, actin cytoskeleton organization, and regulation of cell death (Fig. 1F). In contrast, F4/80^+^ IL1β^+^ MΦs were enriched in inflammatory networks such as response to lipopolysaccharide, NF-κB signaling pathway, and TNF signaling pathway. Separately, F4/80^−^ MHCII^HI^ MΦs exhibited upregulated pathways associated with cellular locomotion and chemotaxis, as well as oxidative phosphorylation and electron transport chain pathways (Fig. 1F). Thus at steady state, we newly define 4 resident macrophage populations derived from contrasting ontogenies that perform distinct immune functions within the peritoneum.

### Internalized apoptotic RNA dissipates after 6 h

**Figure 2:**
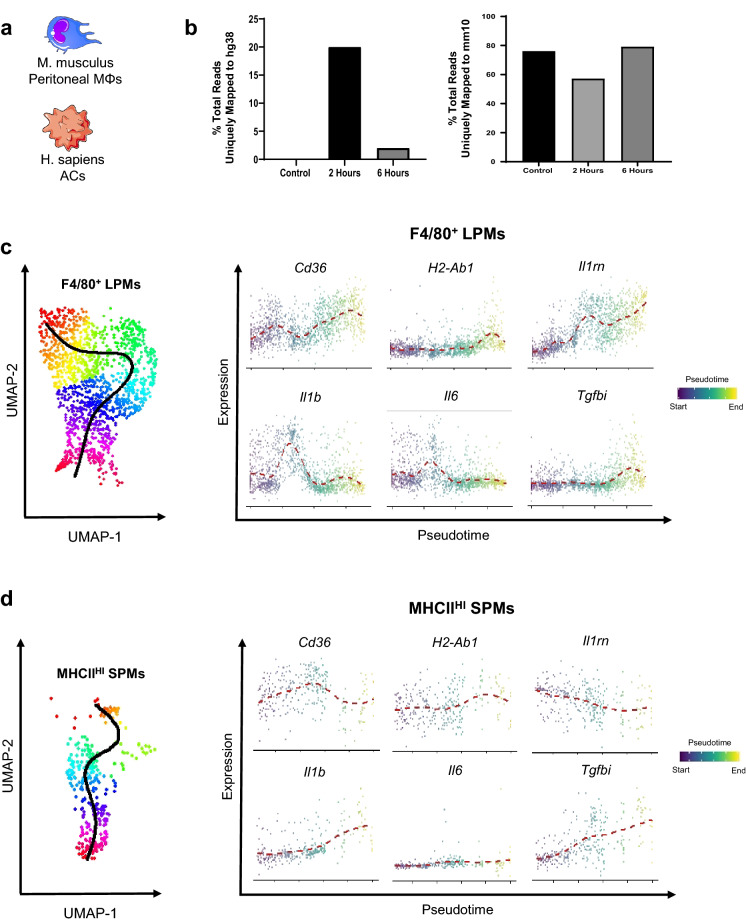
Single-cell pseudotime trajectories reveal dynamic fluctuations of distinct gene expression between F4/80^+^ LPMs and MHCII^HI^ SPMs. (**a**) Resident macrophages (n = 3 per condition) were cultivated with early apoptotic cells (ACs). Non-engulfed cells were removed from adherent phagocytes and transcriptional analysis was performed on 1,800 individual macrophages using the 10 × Genomics pipeline. The figure was produced, in part, by using Servier Medical Art https://smart.servier.com/. (**b**) Quantification of human (hg38) and murine (mm10) aligned reads. (**c**) Slingshot pseudotime trajectory of F4/80^+^ LPMs and log-fold expression of select genes throughout pseudotime trajectory. Cells are colored by pseudotime. (**d**) MHCII^HI^ SPMs throughout engulfment shown on UMAP and log-fold expression of select genes throughout pseudotime trajectory. Cells are colored by pseudotime.

To ascertain the degree of transcriptional heterogeneity of the efferocytic response, we employed a strategy whereby murine peritoneal MΦs were co-cultivated with human Jurkat T cells expressing the GFP transgene under the native Survivin promoter (Fig. 2A). The difference in species between the two cell types was chosen in order to track human-specific transcripts, such as *APOL1*, that would mark “passenger” transcripts from the apoptotic cell and therefore permit us to catalog specifically the macrophage transcriptional response. The GFP transgene, which is not naturally expressed in either the mouse or human genome, provided an additional reporter of the apoptotic cell transcriptome. Moreover, the GFP fluorescence also served as a reporter to confirm apoptotic cell engulfment (Supplementary Fig. S2). As depicted in Fig. 2B, the percent total aligned reads uniquely mapping to the human genome (hg38) within peritoneal immune cells increased 2 h after co-cultivation of human apoptotic cells, with a commensurate decrease in the percentage of aligned reads mapping to the mouse genome (mm10). These reads represent the captured and analyzed portion of the transcriptome during acute exposure to apoptotic cells. Unaligned reads, which can indicate the fraction of a library with lower quality sequencing or increased RNA degradation^22^, were then separately mapped to hg38. After 2-h of co-culture, the percent of initially unaligned reads were enriched for sequences uniquely mapping to hg38 (66.1%). This unaligned portion of the library is typically excluded from standard transcriptomic analyses of gene expression. We next mapped this same bin of unaligned reads to *GFP* and *APOL1* sequences in order to assess apoptotic transcripts in the phagocyte that would not have overlapping reads aligning to mm10. The number of raw reads aligning to these sequences was highest (113 raw counts) after 2-h of co-culture (Fig. 2B). According to our defined parameters (as detailed in “Methods”), macrophages without *GFP or APOL1* reads were defined to have reached a stage of efferocytosis in which apoptotic cell mRNAs would likely be degraded. It was at this time point at 6 h that we focused the majority of our transcriptional analysis.

### Single-cell trajectories reveal distinct transcriptional responses between F4/80^+^ and MHCII^HI^ macrophages after efferocytosis

With our inference that each cell represented in our analysis was at a unique stage of efferocytosis, we performed a pseudotemporal reconstruction using Slingshot, a tool that infers global lineage structures and pseudotime variables for each cell along a lineage^23^. Two distinct trajectories were observed which corresponded to unique transcriptional changes in our F4/80^+^ LPMs and MHCII^HI^ SPMs (Fig. 2C,D). Gene expression was subsequently plotted as a function of pseudotime to track changes across separate MΦ efferocytic states (Fig. 2C,D). While expression of genes such as *Cd36* and *H2-Ab1* increased as a function of pseudotime in both MΦ subsets, expression of *Il1b* and *Tgfbi* was unique between LPMs and SPMs. In LPMs, *Il1b* expression is greatly decreased as a function of pseudotime, but in SPMs, *Il1b* expression increases marginally as a function of pseudotime. Furthermore, expression of *Il1rn*, a potent antagonist of the IL1 receptor, was greatly increased in LPMs but decreased in SPMs during efferocytosis (Fig. 2D). These results reveal divergent transcriptional programming of MΦ subpopulations after exposure to apoptotic cells modeled using pseudotime trajectory analyses.

**Figure 3:**
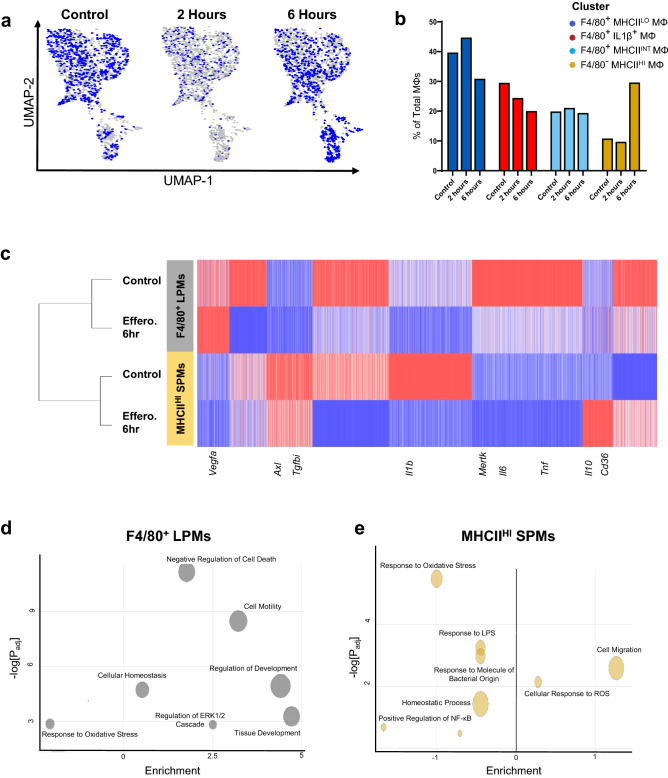
Discrete time points after exposure to apoptotic cells elucidate distinct transcriptional programming among F4/80^+^ LPMs and MHCII^HI^ SPMs. (**a**) UMAP dimension reduction plot of macrophages after co-cultivation of ACs for 2 or 6 h. (**b**) Representative proportion of macrophage clusters in –ACs (Control), + ACs (2 h) or + ACs (6 h) conditions. (**c**) Heatmap of genes clustered using K means clustering (k = 8). Hierarchal clustering was performed using one minus Pearson correlation using Phantasus. (**d**) Gene ontology of differentially expressed genes in F4/80^+^ LPMs and (e) MHCII^HI^ SPMs clusters between –ACs (Control) or + ACs (6 h) conditions was performed using gProfiler. Enrichment is calculated by GOplot and expressed as Z score.

While visualizing gene expression as a function of pseudotime provides insights into how single cell gene expression matures temporally, determining differentially expressed genes remains a statistically difficult challenge^24^. Therefore, we leveraged our physically separated co-culture time points (Fig. 3A,B) to determine differentially expressed genes before and after apoptotic cell exposure. After 6 h of apoptotic cell co-cultivation, F4/80^−^ MHCII^HI^ SPMs are enriched while F4/80^+^ MHCII^LO^ MΦs and F4/80^+^ IL1β^+^ MΦs represent a smaller percentage of the captured MΦ population (Fig. 3B). Genes which had distinct expression among MΦ subsets as a function of pseudotime were confirmed to have similar expressional changes after comparison with data from separate co-culture timepoints (Fig. 3C). Pathway analysis of differentially expressed genes in each MΦ subset before and 6 h after co-culture with apoptotic cells revealed distinct global transcriptional changes after efferocytosis (Fig. 3D,E). Overall, LPMs were enriched for homeostatic and developmental pathways such as tissue development, regulation of development, and cellular homeostasis (Fig. 3D). SPMs, however, uniquely downregulated inflammatory pathways such as response to LPS and positive regulation of NF-κB signaling (Fig. 3E), relative to non-treated MΦs. Interestingly, SPMs also enriched transcription of genes associated with cell proliferation (Fig. 3E), suggesting the increase in SPMs in Fig. 3B may be due to induced cell proliferation. Lastly, to investigate acute transcriptional changes of efferocytosis, gene set enrichment analyses were also performed after 2 h of co-cultivation with ACs. While F4/80^+^ LPMs significantly increased expression of genes for oxidative phosphorylation (Supplemental Fig. S2), MHCII^HI^ SPMs, conversely, significantly reduced expression of genes for oxidative phosphorylation (Supplemental Fig. S2).

### MerTK-deficiency uniquely reprograms F4/80^+^ macrophages versus MHCII^HI^ macrophages

**Figure 4:**
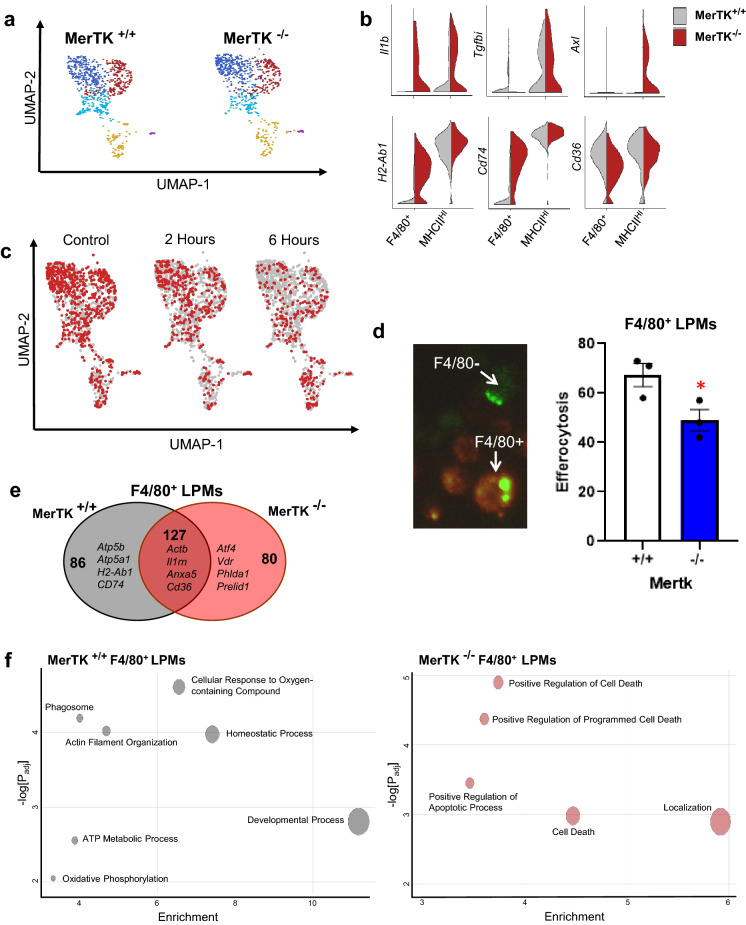
Genetic inactivation of the efferocytosis receptor MerTK impairs efferocytosis programming uniquely in F4/80 macrophages. (**a**) UMAP plots of resident macrophage clusters at steady state between *Mertk*^+/+^ and *Mertk*^*−/−*^ mice. (**b**) Violin Plot of Inflammatory Cytokines and MHCII expression at steady state between F4/80^+^ LPMs and MHCII^HI^ SPMs Clusters. (**c**) UMAP dimension reduction plot of macrophages in *Mertk*^−/−^ mice after co-cultivation of ACs for 2 or 6 h. (**d**) Fluorescently labeled (green calcein-AM) apoptotic Jurkat cells were injected into the peritoneum of *Mertk*^+/+^ versus *Mertk*^*−/−*^ mice and subsequently peritoneal lavages were collected and stained for F4/80^+^ LPMs. Percent double positive F4/80^+^ calcein-AM^+^ cells as function of total F4/80^+^ cells were enumerated. *p < 0.03. (**e**) Venn diagram comparing similar and unique differentially expressed genes. (**f**) Gene Ontology of unique differentially expressed genes in F4/80^+^ LPMs of *Mertk*^+/+^ versus *Mertk*^*−/−*^ mice using gProfiler and GOplot.

To validate that our approach was reporting on consequences of efferocytosis, we incorporated a well characterized efferocytosis receptor knockout, *Mertk*^−/−^^25^. At steady-state, there were no discernible differences of macrophage cluster representation between *Mertk*^+/+^ and *Mertk*^−/−^ peritoneal macrophages (Fig. 4A, Supplemental Fig. S3), consistent with MerTK not being required for the emergence of these cellular populations. However, there were distinct differences in cytokine and MHC allele expression, particularly in the F4/80^+^ LPMs. Specifically, *Mertk*^−/−^ LPMs adopted a pro-inflammatory phenotype compared to *Mertk*^+/+^ LPMs, at steady state (Fig. 4B, Supplemental Fig. S3). This shift in phenotype manifested with an increase in basal expression of inflammatory cytokines *Il1b* and *Il6* and a reduction in anti-inflammatory cytokines such as *Tgfbi* (Fig. 4B). Furthermore, inflammatory pathways were enriched in *Mertk*^−/−^ LPMs, suggesting a heightened basal inflammatory state of these macrophages compared to *Mertk*^+/+^ LPMs (Supplemental Fig. S3). We next performed scRNA-seq analysis on *Mertk*^*−/−*^ peritoneal macrophages co-cultured with ACs for 2 or 6 h. We observed a significant decrease in numbers of F4/80^+^ MHCII^LO^ MΦs but not in F4/80^−^ MHCII^HI^ MΦs 6-h post efferocytosis (Fig. 4C). Additionally, efferocytosis is initially impaired in F4/80^+^ LPMs in *Mertk*^−/−^ mice compared to *Mertk*^+/+^ LPMs (Fig. 4D). Differentially expressed genes of LPMs were determined for each genotype after 6 h of co-culture. Similar and unique differentially expressed genes were identified for *Mertk*^+*/*+^ and *Mertk*^*−/−*^ LPMs (Fig. 4E), indicating distinct transcriptional programming contingent on *Mertk* expression. Gene pathway enrichment analysis of differentially expressed genes unique to each genotype was completed using Gene Ontology (GO) biological processes. *Mertk*^+*/*+^ LPMs upregulated pathways for homeostatic and oxidative phosphorylation pathways, while *Mertk*^*−/−*^ LPMs uniquely increased cell death and apoptosis pathways (Fig. 4F).

## Discussion

Using single-cell RNA sequencing to unbiasedly investigate how exposure to apoptotic cells reprograms phagocytes, our findings reveal a heterogeneity of efferocytic transcriptional responses in resident macrophages not previously appreciated by traditional bulk mRNA sequencing approaches. Importantly, our approach was careful to exclude to the best of our ability the contribution of apoptotic cell-derived transcripts. This is particularly important as low abundant “passenger” RNAs from apoptotic cells could misrepresent the phagocyte transcriptional response, as previously reported^19^.

Our analysis uncovered four transcriptionally distinct phagocyte populations and defined their unique gene expression signatures after co-cultivation of apoptotic cells. A subpopulation of LPMs (F4/80^+^ MHCII^LO^) was enriched for *Klf2 *^21^*, Timd4*^26^*,* and *Mertk*^26^*,* implicating a predisposition for efferocytosis, as these genes encode known regulators of apoptotic cell clearance. Furthermore, the zinc finger transcription factor GATA-binding protein 6 (*Gata6*), a necessary regulator of peritoneum-specific gene expression program in LPMs^15^, is solely expressed in this subset. Separately, a population of SPMs (F4/80^−^ MHCII^HI^) solely expressed the transcription factor *Irf4*, a necessary factor for macrophage differentiation from monocytes^13^. Further validation by gene ontology revealed enrichment for taxis and locomotion pathways, consistent with a monocytic origin of MHCII^HI^ SPMs. Separately, F4/80^+^ MHCII^LO^ MΦs are enriched for developmental and regenerative gene ontology pathways compared to their F4/80^−^ MHCII^HI^ MΦ counterparts. These distinctions in MΦ function further justify that SPMs and LPMs exhibit specialized functions within the peritoneum, specifically that LPMs perform homeostatic maintenance of the peritoneal cavity^27^. Alternatively SPMs have been shown to promote inflammation particularly after thioglycolate-elicitation. However, our data newly identifies a highly inflammatory LPM population (F4/80^+^ IL1β^+^), a population that may have been unidentifiable in previous studies of bulk MΦs. Whether this inflammatory LPM population is the key driver of inflammation within the physiological peritoneal cavity warrants further investigation. Overall, defining differences between ontogenetically divergent MΦs may elucidate mechanisms by which resident peritoneal MΦs uniquely contribute to tissue homeostasis and physiological inflammation.

Upon challenge with apoptotic cells, macrophage subpopulations induced unique transcriptional responses, consistent with the premise that macrophage ontogeny predisposes their response to apoptotic cells. Previously, Cain et al. determined that LPMs are more efficient at efferocytosis than SPMs, but importantly both subsets exhibited apoptotic cell engulfment one hour after exposure^28^. Furthermore, the vast majority of apoptotic cell-derived transcripts are degraded after 6 h of co-cultivation, and thus no phagocytes were removed from the downstream transcriptional analyses at this time point. Consequently, this experimental design does not exclude “late-eater” phagocytes that may have initiated apoptotic engulfment at a later time. Of note, acute transcriptional changes in macrophages after 2 h of co-cultivation are subtle relative to transcriptional responses 6 h after co-cultivation. This observation is consistent with the paradigm that acute phagocyte responses activate post-transcriptional cytoskeletal remodeling, while phagocyte transcriptional reprogramming is a subsequent event. Therefore to better model the transcriptional landscape throughout differential phases of efferocytosis, we employed a pseudotime trajectory analysis using Slingshot. Since these algorithms rely on transcriptional differences independent of time to produce a pseudotime trajectory, the modeled response and reprogramming trajectories during efferocytosis are better synchronized to allow for comparison between macrophage clusters. Unsurprisingly, both LPMs and SPMs revealed distinct transcriptional programs upon exposure to apoptotic cells. LPMs robustly reduced their inflammatory gene expression as a function of pseudotime, suggesting that exposure to apoptotic cells polarizes LPMs towards an anti-inflammatory state. Furthermore, many of the differentially expressed genes in LPMs after apoptotic cell exposure for 6 h are enriched in homeostatic and developmental pathways. These data are consistent with apoptotic cell clearance by LPMs as an essential mechanism for tissue homeostasis. Conversely, SPMs reduce enrichment of inflammatory pathways such as NF-kB signaling and response to LPS, while increase enrichment of cell proliferation pathways after apoptotic cell exposure (Fig. 3E). These results may lead to the observation of an increase in MHCII^HI^ SPMs after 6 h of AC co-cultivation. Our careful approach using pseudotime algorithms combined with discrete time points allow us to compare the transcriptional changes during efferocytosis between the distinct macrophage populations.

We also leveraged macrophages deficient in *Mertk,* a key efferocytosis receptor^25^ to validate and investigate the effects of impaired apoptotic cell clearance on peritoneal macrophage subsets. *Mertk* is primarily expressed in LPMs and *Mertk-deficient* macrophages exhibited acute reductions in F4/80 + macrophages (Fig. 4D). At steady-state, *Mertk-*deficient LPMs exhibited significantly increased MHCII expression and pro-inflammatory cytokine expression. Upon challenge with apoptotic cells, *Mertk*^*−/−*^ LPMs exhibited a signature of programmed cell death (Fig. 4F). These data are consistent with previous studies implicating *Mertk* as a mediator of cell survival after the stress of efferocytosis^29–31^. Intriguingly, expression of *Mertk* is associated with promoting phagocyte survival after exposure to oxidative stress through the upregulation of anti-apoptotic signals including pAkt and pErk^29^. Previously it has been shown that Abca1 reverse cholesterol efflux pathway dampens oxidative stress preserving phagocyte viability after exposure to apoptotic cells or oxidized phospholipids^32^. Without the beneficial role of Abca1 reverse cholesterol efflux, engulfment of apoptotic cells induced an excessive oxidative burst leading to activation of the apoptotic cell death program. Therefore, our findings suggest that *Mertk*-expression specifically induces cellular homeostatic responses to oxidative stress in LPMs that preserve phagocyte viability after efferocytosis.

Future studies are warranted to examine the heterogeneity of efferocytic transcriptional responses in situ and in vivo. This could be achieved by coupling single cell-sequencing approaches with tissue phagocytes that are positive for the engulfment of reporter apoptotic cells. However, under these conditions, it would be difficult to exclude the contribution of apoptotic cell-derived transcripts. One potential circumvention would be the employment of Ribotag technology^33^ to capture newly formed transcripts in the phagocyte. While scRNAseq has emerged as a powerful and insightful technique, additional studies are also needed to validate the transcriptional signatures of our findings at the protein level, possibly through technologies such as CyTOF or high-dimensional flow cytometry. With these future directions in mind, our studies newly elucidate the heterogenous transcriptional response of single-cell peritoneal macrophages, after exposure to apoptotic cells. These findings will benefit future studies that seek to determine the unique functional roles of phagocyte subsets during efferocytosis.

## Methods

### Animal studies

C57BL/6J mice were used as WT controls and bred in the Northwestern Center for Comparative Medicine facility. *MerKD* (referred to herein as *MerTK*^*−/−*^) have been previously described^25^ and were backcrossed to C57BL/6J mice for ten generations. Eight- to twelve- week old mice were utilized for experiments. Mice were bred and housed in a pathogen-free, temperature- and humidity-controlled environment with access to standard mouse chow and water ad libitum. Mice were kept on a 12:12 light/dark cycle. All studies were approved and reviewed by the Institutional Animal Care and Use Committee at Northwestern University (Chicago, Illinois), protocol #IS00000378. All experiments were performed in accordance with the guidelines and regulations named in the protocol.

### Ex vivo efferocytosis assay, microscopy, and flow cytometry

Resident peritoneal cells were harvested after lavage with cold saline. Peritoneal cells from 3 mice were pooled together for each experimental group. Initial cell selection was achieved through adherence to non-treated, low-adherence cell culture plates for 1 h and rinsed to remove non-adherent cells. Apoptotic cells (ACs) were generated using GFP-Jurkat Human T cells (GenTarget) exposed to UV radiation for 7 min followed by a 2-h incubation at 37 °C. Apoptosis was confirmed by annexin V positive, propidium iodide negative identification affirming greater than 80% apoptosis. Adherent resident peritoneal cells were co-cultivated with ACs at a ratio of 5 ACs to 1 peritoneal cell for 2 h or 6 h as indicated. Control cells were given a media change corresponding to the 6-h timepoint. Non-engulfed ACs were removed from the co-culture through rigorous rinsing with warm saline. Adherent macrophages were removed from the plate with Accutase (StemCell Technologies) and resuspended into a single cell suspension. Apoptotic cell engulfment was confirmed with confocal fluorescence microscopy.

For in vivo efferocytosis microscopy, fluorescently labelled (green calcein-AM) apoptotic Jurkat cells were injected into the peritoneum of Mertk^+/+^ versus Mertk^−/−^ mice and subsequently peritoneal lavages were collected and stained for F4/80 + resident macrophages. Percent double positive F4/80 + calcein-AM + cells as function of total F4/80 + cells were enumerated. *p < 0.03.

### Single cell library preparation and RNA sequencing

The single cell RNA-Seq libraries were prepared using the 10X Genomics Single Cell 5′ Gel Bead and Library Kit pipeline following manufacturer’s protocols. Cell suspensions were diluted to target a recovery of 4,000 cells per sample. A total of 7,674 resident peritoneal cells were sequenced to a read depth ~ 100,000 reads per cell. The Illumina libraries were run on an Agilent Bioanalyzer High Sensitivity Chip and Kapa Library Quantification Kits for Illumina platform (KAPA Biosystems) for quality control before sequencing. In collaboration with the Northwestern University Sequencing Core (NuSeq), the libraries were sequenced on the Illumina HiSeq 4,000 with the following parameters: Read 1—26 cycles; i7 Index—8 cycles; Read 2—98 cycles.

### Cell ranger, read alignment, and quantifying cells with ‘unaligned reads’

The sequenced data were processed with the Cell Ranger Single Cell software suite 1.3.1 by 10 × Genomics (GEO accession number pending). Briefly, raw base-call files from a HiSeq4000 sequencer were demultiplexed into FASTQ files. FASTQ files from each of the samples were mapped and the genes were counted using *cellranger* count. Given that the sequencing parameters included paired-end reads and a full length (150 bp) second read, the “SC5P-PE” option was used for the *chemistry* parameter. The sequencing reads were aligned to both *hg38* (Homo sapiens) and *mm10* (Mus musculus) reference genomes using STAR aligner. Reads mapped to *mm10* were used for downstream analysis with Seurat.

The unaligned reads were extracted from the ‘possorted_bam.bam’ file created by CellRanger using Samtools with the ‘-f 4’ flag. Awk was then used to remove any duplicate reads with the ‘!seen[$0] +’ distinction. The unmapped read ids were then used as a pattern to grep the FASTQ reads from the raw FASTQ file with ‘seqkit’ for both read one and two. These reads were then mapped to a custom genome containing only the APOL1 genes using the STAR aligner. After alignment, the mapped reads were extracted using Samtools view with the ‘-F 4’ flag and deduplicated using ‘awk’ with the ‘!seen[$0]++’ distinction. For the purpose of determining the number of filtered cells containing the human DNA; the mapped read ids were grepped by ‘seqkit’ to extract the reads from the raw fastq files that had been mapped to APOL1 followed by the 10 × cell barcodes being identified using ‘sed’ with the ‘2 ~ 4p’ distinction for the ‘-n’ parameter. Lastly, the 10 × barcodes from the Seurat object were extracted and compared using ‘comm’ to the read extracted 10X barcodes to count the number of cells containing the human APOL1 DNA.

### Single-cell RNA sequencing analysis and visualization

The barcode-gene matrices from the Cell Ranger pipeline were further analyzed using the Seurat R package (v.3.1)^34^. Following standard practices to exclude low-quality cells, cells that expressed less than 500 genes, had a ratio of unique molecular identifiers (UMIs) per gene below 0.8, or had greater than 10% mitochondrial genes were filtered from the datasets. Furthermore, each cell was assigned a Cell Cycle Score to address potential differences in cell cycle phase.

Normalization and variance stabilization of molecular count data was carried out using the R package *sctransform* (v0.2.1)^35^. After transformation, each sample was integrated using Seurat’s modified integration workflow to harmonize the output Pearson residuals across datasets. To reduce the dimensionality of the expression matrices, we performed a principal component analysis (PCA). The number of principle components for further downstream applications was decided to be 40, as UMAP was employed for dimensionality reduction and visualization of the data.

Clusters were identified using graph-based clustering approach implemented by the FindCluster function in Seurat at resolutions of 0.2, 0.4, 0.6, 0.8, and 1.0 to determine transcriptionally distinct populations of resident peritoneal cells. Clusters with high cell cycle scores were excluded from further downstream analyses. To unbiasedly identify resident peritoneal cells present in the dataset, *SingleR* (v1.0.5) was employed. Briefly, *SingleR* infers the origin of each individual cell by referencing transcriptomic datasets of pure cell types. We utilized the ImmGen database, which contains normalized expression values for immune cells from 830 murine microarrays to ID our peritoneal cell types. These classifications were confirmed with canonical immune cell markers.

For all differential expression tests, we utilized the model-based analysis of single-cell transcriptomics (MAST) test in the Seurat package. To identify unique expression profiles for each cluster, differential expression was tested between each macrophage cluster and all other macrophage clusters combined. The top 50 differentially expressed genes (based on average log fold change) unique to each cluster were visualized in a heatmap. Differential expression testing was also performed between two individual clusters, between genotypes, and between timepoints.

### Pathway enrichment analysis

To identify enriched molecular pathways based on differentially expressed genes, gProfiler^36^ was applied. Gene sets from Gene Ontology biological process and Kyoto Encyclopedia of Genes and Genomes (KEGG) were used. GOplot (v1.0.2) was employed for visualization of the GO enrichment analyses.

### Single-cell trajectory inference

Slingshot (v1.4.0)^23^ was applied to infer efferocytosis reprograming trajectories of macrophages which experienced apoptotic cell engulfment. Slingshot combines highly stable techniques to infer novel lineages of the global structure. Each macrophage subpopulation (F4/80 + and MHCII +) were isolated into separate single-cell experiments and reduced dimensionality via both PCA and UMAP. The UMAP trajectories were utilized for visualization purposes, while the PCA trajectories were utilized for gene expression plots as UMAP dimensionality reduction has been shown to distort the pseudotime data^37^. Changes in gene expression as a function of pseudotime were visualized using the PlotExpression command.

## Supplementary information

Supplementary file 1

## Data Availability

The datasets generated during and/or analyzed during the current study are available from the corresponding author on reasonable request. The datasets are available in the National Center for Biotechnology Information Gene Expression Omnibus (GEO) repository (GSE156234).

## Code availability

R scripts are available through github (https://github.com/thorplab/Macrophage-Efferocytosis-Single-Cell). There is no restriction on the use of the code or data.
