# Supplementary material for: Single-cell RNA sequencing uncovers heterogenous transcriptional signatures in macrophages during efferocytosis
Source: Sci Rep. 2020 Aug 31;10:14333. doi: 10.1038/s41598-020-70353-y (PMC7459098; doi:10.1038/s41598-020-70353-y)
Supplement: Supplementary file 1 [file 41598_2020_70353_MOESM1_ESM.pdf]

Single-Cell RNA Sequencing Uncovers Heterogenous Transcriptional Signatures in Macrophages during Efferocytosis

Connor Lantz*[1], Behram Radmanesh[1], Esther Liu[1], Edward B. Thorp#[1], Jennie Lin#[1,2]

*Corresponding Author (connor.lantz@northwestern.edu)

#Co-Senior Authors

[1]Northwestern University, Feinberg School of Medicine, Departments of Pathology, Pediatrics, Medicine and the Feinberg Cardiovascular and Renal Research Institute,

[2]Jesse Brown Veterans Affairs Medical Center, Chicago IL, 60611

Supplemental Figure 1

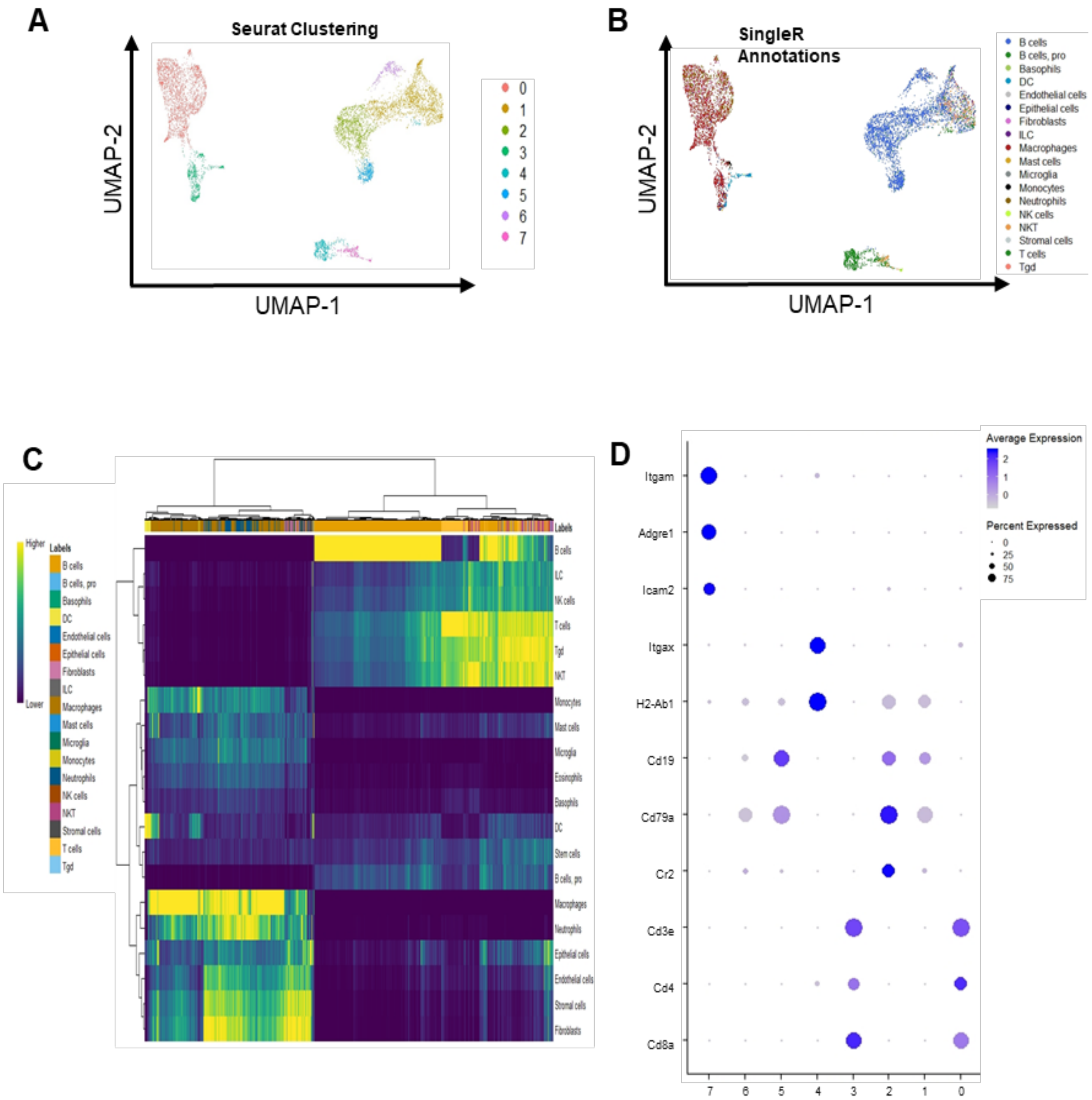

Supplemental Figure 1 – Unbiased identification of resident peritoneal immune subsets using SingleR and Immgen's open-sourced reference database

(A) Unbiased clustering of resident peritoneal immune cells at a resolution of 0.2 performed by Seurat

(B) SingleR annotations of cell identity generated through comparison to Immgen's reference database

(C) Heatmap of SingleR scores for all cells across all reference labels

(D) Dot plot of traditional immune marker expression within each Seurat cluster from (A)

Supplemental Figure 2

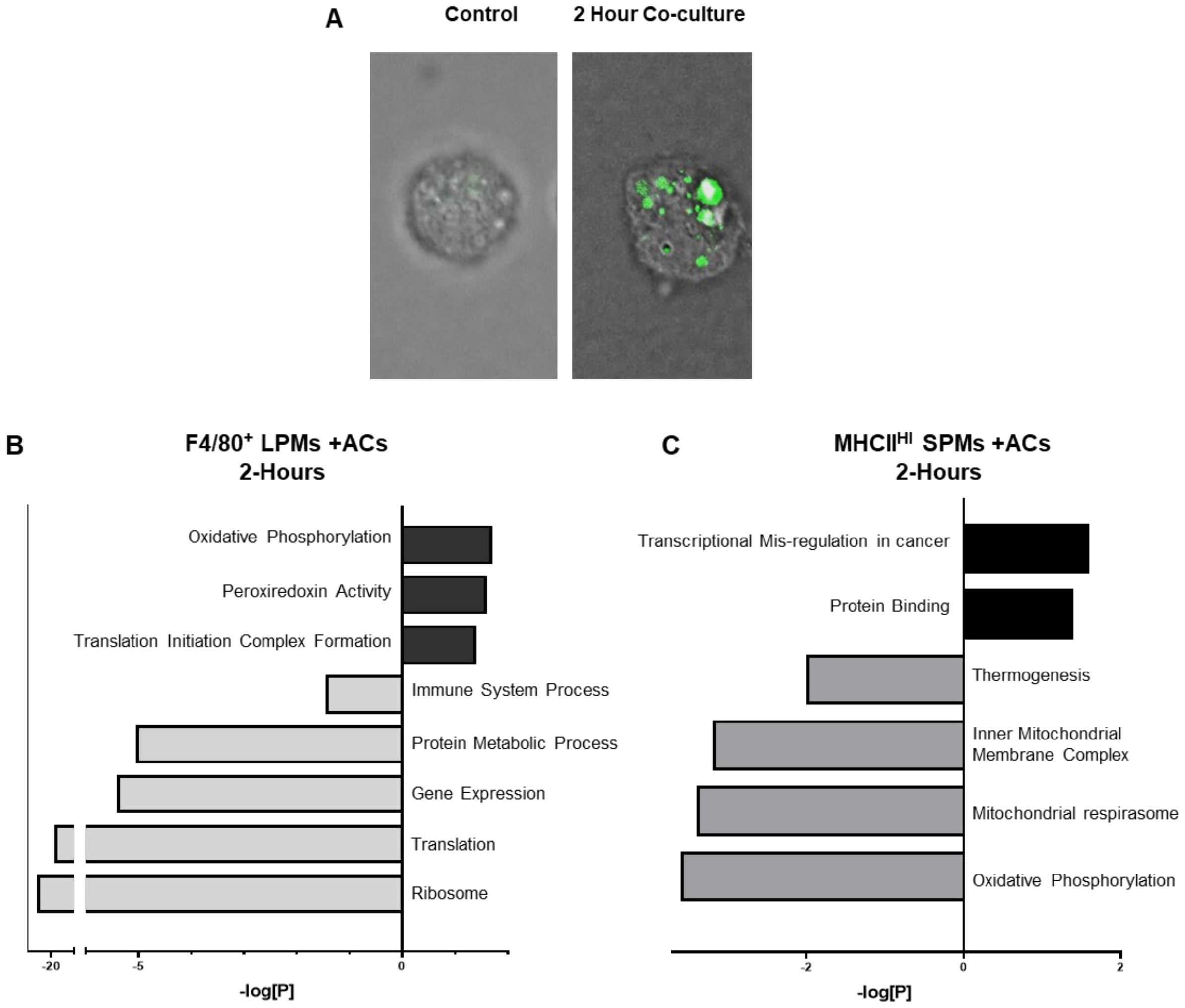

Supplemental Figure 2 – Apoptotic Jurkat Cells are engulfed by peritoneal phagocytes after 2 hours co-cultivation and induce acute transcriptional programming

(A) Fluorescence microscopy of resident peritoneal macrophage engulfment of GFP-labeled apoptotic T cells ex vivo during 2 hours of co-cultivation

(B) Gene ontology of differentially expressed genes in F4/80$^+$ LPMs between –ACs (Control) or +ACs (2hour) conditions was performed using gProfiler.

(C) Gene ontology of differentially expressed genes in MHCII^{HI} SPMs between –ACs (Control) or +ACs (2hour) conditions was performed using gProfiler.

Supplemental Figure 3

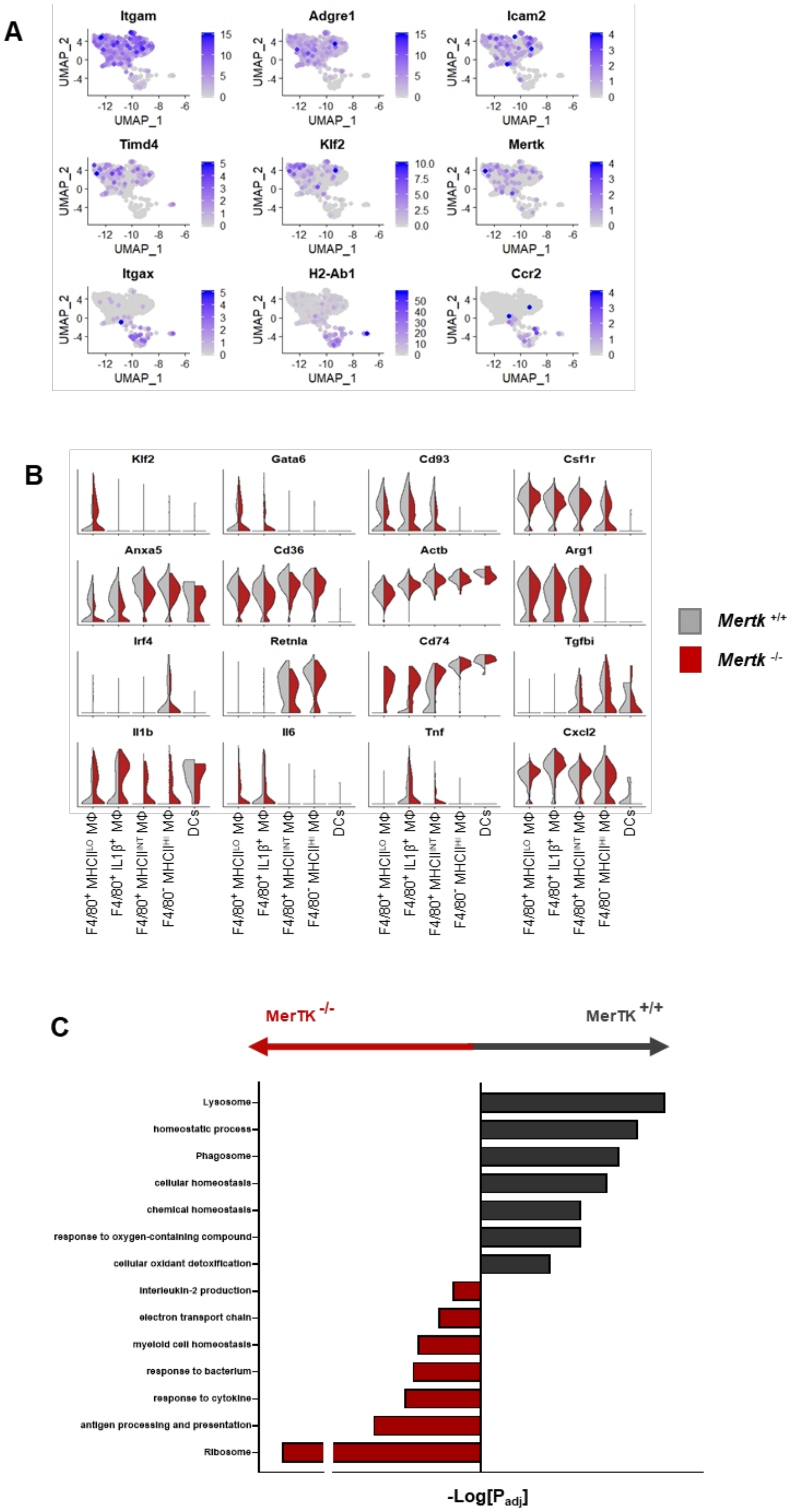

Supplemental Figure 3 – *Mertk* $^{-/-}$ MΦs exhibit similar ontogeny markers but have a higher basal inflammation state compared to *Mertk* $^{+/+}$ MΦs at steady state

(A) Gene expression patterns representing single-cell gene expression of canonical peritoneal macrophage markers in *Mertk* $^{-/-}$ MΦs

(B) Violin plots revealing distinct expression of macrophage differentiation mediators and inflammatory genes between *Mertk* $^{+/+}$ and *Mertk* $^{-/-}$

(C) Gene Ontology of differentially expressed genes in F4/80^{+} LPMs cluster comparing between *Mertk* $^{+/+}$ and *Mertk* $^{-/-}$ at steady state using gProfiler. Pathway enrichment is expressed as the –log[p.value] adjusted for multiple comparisons
